# FTY720/Fingolimod mitigates paclitaxel‐induced Sparcl1‐driven neuropathic pain and breast cancer progression

**DOI:** 10.1096/fj.202401277R

**Published:** 2024-08-10

**Authors:** Sandeep K. Singh, Cynthia Weigel, Ryan D. R. Brown, Christopher D. Green, Connor Tuck, Daniela Salvemini, Sarah Spiegel

**Affiliations:** ^1^ Department of Biochemistry and Molecular Biology Virginia Commonwealth University School of Medicine Richmond Virginia USA; ^2^ Department of Pharmacology and Physiology School of Medicine and Institute for Translational Neuroscience Saint Louis University St. Louis Missouri USA

**Keywords:** breast cancer, CIPN, paclitaxel, sphingosine‐1‐phosphate receptor 1

## Abstract

Paclitaxel is among the most active chemotherapy drugs for the aggressive triple negative breast cancer (TNBC). Unfortunately, it often induces painful peripheral neuropathy (CIPN), a major debilitating side effect. Here we demonstrate that in naive and breast tumor‐bearing immunocompetent mice, a clinically relevant dose of FTY720/Fingolimod that targets sphingosine‐1‐phosphate receptor 1 (S1PR1), alleviated paclitaxel‐induced neuropathic pain. FTY720 also significantly attenuated paclitaxel‐stimulated glial fibrillary acidic protein (GFAP), a marker for activated astrocytes, and expression of the astrocyte‐secreted synaptogenic protein Sparcl1/Hevin, a key regulator of synapse formation. Notably, the formation of excitatory synapses containing VGluT2 in the spinal cord dorsal horn induced by paclitaxel was also inhibited by FTY720 treatment, supporting the involvement of astrocytes and Sparcl1 in CIPN. Furthermore, in this TNBC mouse model that mimics human breast cancer, FTY720 administration also enhanced the anti‐tumor effects of paclitaxel, leading to reduced tumor progression and lung metastasis. Taken together, our findings suggest that targeting the S1P/S1PR1 axis with FTY720 is a multipronged approach that holds promise as a therapeutic strategy for alleviating both CIPN and enhancing the efficacy of chemotherapy in TNBC treatment.

## Graphical abstract

Triple‐negative breast cancer (TNBC) is aggressive and has limited therapeutic options. Paclitaxel is an effective chemotherapy; however, it often induces peripheral neuropathy (CIPN), a debilitating side effect. In naive and breast tumor‐bearing immunocompetent mice, FTY720/Fingolimod that targets S1PR1, suppressed paclitaxel‐induced neuropathic pain, astrocyte activation, the astrocyte‐secreted synaptogenic protein Sparcl1/Hevin and synapse formation. FTY720 also enhanced the anti‐tumor effects of paclitaxel. Thus, FTY720 is a multipronged approach that holds promise for alleviating CIPN and enhancing the efficacy of chemotherapy in TNBC treatment.

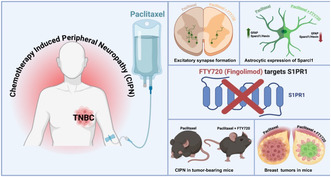

**Abbreviations**
CIPN: chemotherapy‐induced peripheral neuropathy
DH‐SC: dorsal horn of the spinal cord
GFAP: glial fibrillary acidic protein
NMDARs: glutamate N‐methyl‐D‐aspartate receptors
S1P: sphingosine‐1‐phosphate
S1PR1: sphingosine‐1‐phosphate receptor 1
SphK: sphingosine kinase
TNBC: triple negative breast cancer

## 1 INTRODUCTION

Triple‐negative breast cancer (TNBC) is aggressive, resistant to hormonal therapy, and prone to metastasis to other organs with few therapeutic options.^1^ Targeted therapies are not very effective and most TNBC patients rely mainly on conventional chemotherapy. Paclitaxel that disrupts cytoskeletal microtubules is among the most active chemotherapy drugs for TNBC that prolongs survival.^2^, ^3^ Unfortunately, many patients receiving paclitaxel develop chronic, distal, and symmetrical sensory peripheral neuropathy, often accompanied by a neuropathic pain syndrome known as chemotherapy‐induced painful peripheral neuropathy (CIPN).

The pathophysiological processes underlying CIPN are related to damage to peripheral nerves, increased‐neuronal sensitivity, NMDA receptor function, synapse number, and glial cell‐mediated neuroinflammation^4^, ^5^; however, the precise cellular and molecular mechanisms of CIPN remain poorly characterized. Preclinical studies suggest that CIPN arises from the dysregulation of sphingolipid metabolism in the dorsal horn of the spinal cord (DH‐SC) and increased production of the bioactive sphingolipid metabolite sphingosine‐1‐phosphate (S1P).^6^, ^7^, ^8^, ^9^, ^10^ Alterations in sphingolipid metabolism, particularly increased sphinganine‐1‐phosphate, also have been observed in breast cancer patients with paclitaxel‐induced neuropathy.^11^ S1P has also been implicated in TNBC progression and metastasis.^12^, ^13^, ^14^, ^15^ S1P is formed by sphingosine kinases (SphK1 and SphK2) and acts mainly by binding to five G protein‐coupled receptors, called S1PR1–5.^16^ In particular, the S1P/S1PR1 axis was shown to activate a feed forward amplification loop promoting TNBC growth and metastasis.^13^ On astrocytes, the S1P/S1PR1 axis activates NLRP3 inflammasome and release of IL‐1β, leading to neuroinflammation, altered glutamatergic homeostasis, and reduced neuronal excitability, processes contributing to the development of CIPN.^7^, ^8^, ^9^, ^10^

Activation of astrocytes and their interaction with neurons is involved in the development of neuropathic pain, characterized by astrocyte secretion of pro‐inflammatory cytokines, modifications in spinal synapses, and modulation of synaptic activity.^17^, ^18^, ^19^ Previously, we have shown that the astrocyte‐secreted synaptogenic protein Sparcl1 (also known as Hevin), recruits glutamate N‐methyl‐D‐aspartate receptors (NMDARs) to the synapse and is required for synaptic formation and plasticity.^20^ Like S1P,^21^ intrathecal injection of Sparcl1 induced persistent mechanical allodynia in mice.^22^ Sparcl1 levels are increased in the cerebrospinal fluid of nerve‐injured mice and after plantar incision‐induced postoperative pain.^22^, ^23^ However, the role of Sparcl1 in CIPN and its relationship to the S1P/S1PR1 axis in astrocytes is still unclear.

Targeting S1PR1 with the pro‐drug FTY720 (Fingolimod), that is phosphorylated in vivo by SphK2 to a S1P mimetic and acts as a functional S1PR1 antagonist, suppresses TNBC growth and metastasis.^13^, ^24^, ^25^ Other reports showed that FTY720 is also effective at reducing CIPN.^8^, ^9^, ^10^ The majority of CIPN studies have been carried out in naïve mice and only very few studies have been performed in tumor‐bearing animals, yet some clinical data suggest that the presence of tumors may contribute to the behavioral phenotype associated with neuropathic pain.^26^ Thus, in this study, we have investigated the role of S1P/S1PR1 and Sparcl1 in the development of paclitaxel‐induced CIPN in a syngeneic mouse model of TNBC. We also examined the hypothesis that targeting the S1P/S1PR1 axis with FTY720 is a multi‐pronged approach to enhance the anti‐cancer effects of paclitaxel while suppressing CIPN.

## 2 MATERIALS AND METHODS

### 2.1 Study approval

The Institutional Animal Care and Use Committee of Virginia Commonwealth University approved all animal protocols and procedures. Eight‐week‐old female C57Bl/6J mice (#000664) obtained from Jackson Laboratory (Bar Harbor, ME # 000664) were housed in the animal care facilities at the Virginia Commonwealth University under standard temperature, humidity, and timed light conditions and provided with standard rodent chow and water ad libitum. Mice were acclimatized for about a week in the VCU housing facility before performing any experiment such as mechanical allodynia, cold allodynia, or tumor cell implantation.

### 2.2 Mechanical allodynia measurement

Unrestrained mice were placed in wire mesh baskets and covered with dark cloth and allowed 45 min to 1 h to settle before mechanical allodynia was assessed with von Frey filaments. Briefly, von Frey filaments were applied randomly to the left and right plantar surfaces of the hind paw as described.^6^, ^7^ Lifting, licking, or shaking of the paw in response to three stimulations was recorded as positive responses. Once a positive response was detected, sequentially lower weight filaments were used to assess the sensory threshold for each paw. Basal von Frey paw withdrawal response was assessed 4–5 days prior to tumor cell implant and 25 days post tumor cell implant. Sensory threshold of paw withdrawal was combined and averaged for both left and right paw from each mouse.

### 2.3 Cold allodynia measurement

Unrestrained mice were placed in a wire mess basket and the cold allodynia was assessed with acetone application test.^27^ Briefly, 10 μL acetone was shot randomly on the right or left plantar surfaces of the hind paw. Time duration of lifting, licking, or shaking of the paw in response to acetone was counted by stop/start timer. This response was measured for a total 1‐min duration per paw. Sensory threshold of cold allodynia was combined and averaged for both left and right paw from each mouse. Cold allodynia measurement was taken 2 h post mechanical allodynia.

### 2.4 Treatment of tumor‐bearing mice with paclitaxel and FTY720

To investigate tumor progression and lung metastasis, 8‐week‐old female C57Bl/6J mice obtained from Jackson Laboratory (Bar Harbor, ME # 000664) were bilaterally implanted in the 4th mammary fat pads with 20 μL of DMEM (glucose‐ and phenol red‐free, Gibco #A14430‐01) containing 0.1 × 10^6^ E0771.LMB murine breast cancer cells. Tumor growth was monitored by measuring palpable tumor size with calipers, and volume was computed using the formula *V* = (*W* ^2^× *L*)/2, where *V* represents tumor volume, *W* stands for tumor width, and *L* denotes tumor length. Starting at day 12 after tumor plantation when tumors were palpable, mice were treated every other day with vehicles, FTY720 by gavage (0.3 mg/kg diluted in saline), paclitaxel (i.p. 10 mg/kg diluted in saline), or both. Paclitaxel was obtained from Athenex (6 mg/mL paclitaxel in 527 mg polyoxyl 35 castor oil, 48.7% (v/v) dehydrated alcohol, 2 mg citric acid). Pain measurements were made prior to tumor cell implantation and on day 25 as described above. At the end of experiment, mice were sacrificed by 5% isoflurane inhalation, blood was collected, mice were perfused transcardially with PBS containing 0.1% heparin, tissues were removed for analyses, tumors, and lungs as well as were excised and weighed, fixed in 4% paraformaldehyde (PFA), and embedded in paraffin or frozen in liquid nitrogen. Lumbar regions of spinal cords were also immediately embedded in 4% PFA.

### 2.5 Immunofluorescence

Spinal cords (SCs) were post‐fixed in 4% PFA for a week at 4°C and then cryopreserved in 30% sucrose (in 1x TBS) for 2–3 days at 4°C. Sucrose‐immersed SCs then were embedded in an OCT medium. Ten micrometer sections were prepared on glass slides using a cryostat (Thermo‐Fisher). Sections were washed three times in TBS (25 mM Tris‐base, 135 mM NaCl, 3 mM KCl, pH 7.6), then blocked with 10% normal donkey serum and 0.5% Triton‐X100 in TBS for 1 h at room temperature. Sections were incubated overnight at 4°C with the following antibodies in blocking buffer: anti‐GFAP (1:500, Cell Signaling; #3670), anti‐Sparcl1 (1:500, R & D Systems; #AF2836), anti‐PSD95 (1:500, Thermo Fisher; 51‐6900), and anti‐VGLUT2 (1:1000, Millipore; # AB2251). Subsequently, sections were washed and incubated with species‐specific secondary antibodies conjugated with Alexa Fluor 594 or Alexa Flour 488 (1:500, Invitrogen) for 2 h at room temperature, followed by washing with TBS and finally mounted with DAPI containing Vectashield mounting media (Vector Labs #H‐1000‐10). Sections were imaged using Zeiss LSM 710. Multiple confocal images were taken at 20x (GFAP and Sparcl1) or 63X with 1.4x optical zoom (VGLUT2 and PSD95).

### 2.6 Image analyses

Images were captured with Zeiss 880 and Zeiss 710 confocal microscopes at 20x (for GFAP and SPARCL1) or 63x (for synapse analyses). For synapse analyses, confocal images were obtained with 0.35 μm step sizes in 6–8 Z‐stacks, totaling 2–3 μm. Representative images underwent processing for average intensity projections of 1–2 optical steps in FIJI. Multiple images were acquired per mouse. Sparcl1 and GFAP signal intensity was quantified by puncta analyzer plug in using the Image J software and presented as average integrated signal intensity per image. Synapses were quantified as colocalized puncta (yellow) between VGLUT2 (red) and PSD95 (green) signal using Puncta Analyzer plug in tool in Image J. VGLUT2 and PSD95 puncta were quantified similarly. Pearson's correlation coefficients were calculated using Coloc2 plugin for ImageJ.

### 2.7 Quantification and statistical analysis

For the in vivo lung metastasis and primary tumor growth experiments, the number of mice in each group is indicated in the figure legends. Statistical significance was determined using unpaired two‐tailed Student's t‐test with Welch's correction for comparison of two groups unless indicated otherwise, or by ANOVA followed by post hoc tests for multiple comparisons using GraphPad Prism 7.0. For all experiments, the normality of the data from each group was first checked using the Shapiro–Wilk statistical test. For non‐normally distributed data, the Mann–Whitney U test was used. The following designations for significance levels are *p < .05, **p < .01, ***p < .001, and ****p < .0001.

## 3 RESULTS

### 3.1 FTY720 suppresses paclitaxel‐induced increases in Sparcl1 expression and excitatory synapse formation

**FIGURE 1:**
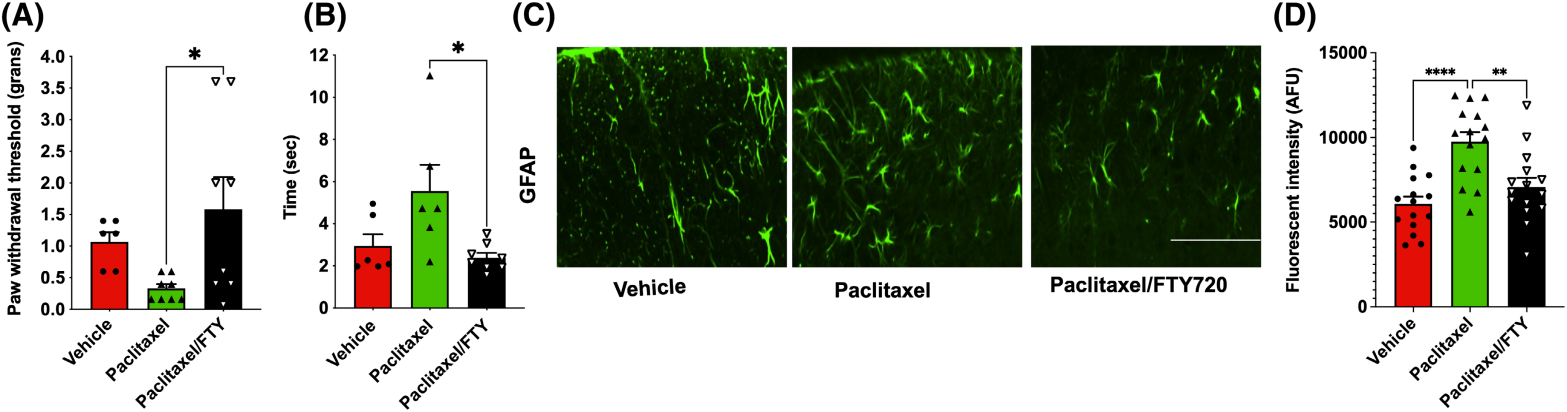
S1PR1 antagonism attenuates mechano‐allodynia. (A–D) Naive C57BL6/J mice were intraperitoneally injected with paclitaxel (2 mg/kg) or vehicle on four alternate days (0, 2, 4, 6). FTY720 (0.3 mg/kg) was orally administered concurrently with paclitaxel. Mechano‐allodynia (A) and cold‐allodynia (B) were measured in each paw on day 15. *n* = 3–4 mice per group. (C and D) FTY720 suppressed paclitaxel‐induced astrocyte reactivation in the DHSC. (C) Representative images (20X) of GFAP immunostained (green) DHSCs from mice treated with vehicle, paclitaxel, or paclitaxel +FTY720‐treated mice. Scale bar: 100 μm. (D) Quantitation of GFAP staining plotted as integrated density per field. *n* = 3 mice per group. Fifteen to sixteen images per group were acquired and quantified. Data are mean ± SEM. *p < .05 **p < .01, ****p < .0001. One‐way analysis of variance test followed by Tukey's multiple comparison test.

Consistent with a previous study,^6^ FTY720 administration to naïve mice significantly blocked mechanical allodynia induced by paclitaxel (Figure 1A) and cold allodynia measured by application of acetone on the hind paw (Figure 1B). We also found that GFAP, a marker for activated astrocytes, was increased by paclitaxel in the dorsal horn of the spinal cord (DHSC) indicating an increase in the number of astrocytes, their activation, or both. Moreover, FTY720 also attenuated paclitaxel‐induced astrocyte activation (Figure 1C,D), supporting the involvement of astrocytes in CIPN as was previously reported.^7^, ^8^, ^9^, ^28^, ^29^

**FIGURE 2:**
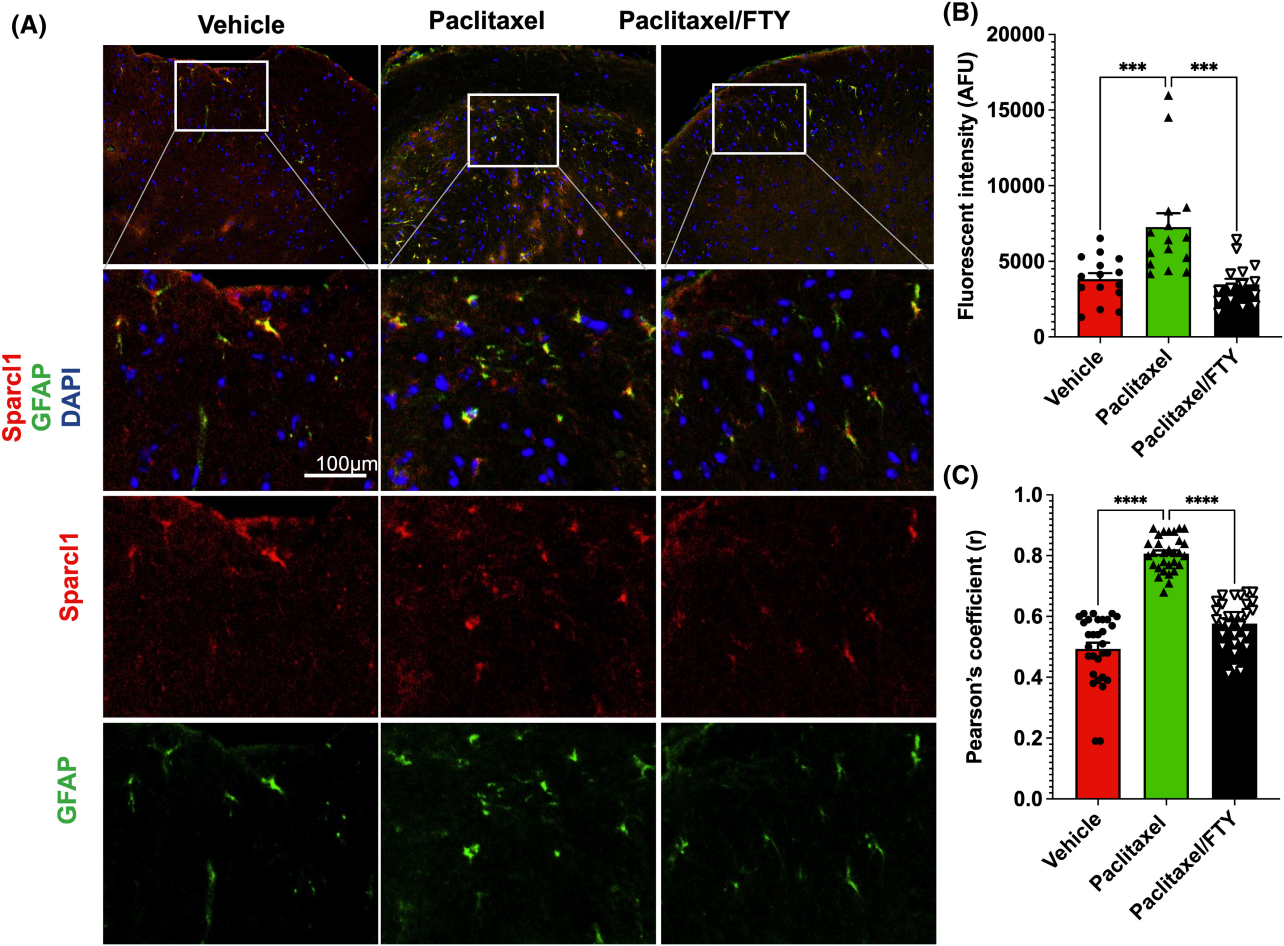
FTY720 suppresses paclitaxel induced Sparcl1 expression in naïve mice. Mice were treated with vehicle, paclitaxel without or with FTY720, as described in Figure 1. (A) Representative confocal images of the dorsal horn spinal cord area immunostained for GFAP (green) and Sparcl1 (red). (B) Quantification of Sparcl1 fluorescence intensity plotted as integrated density per field. *n* = 3 mice per group. Fifteen to sixteen images per group were acquired and quantified. (C) Pearson's correlation coefficient of colocalization between GFAP and Sparcl1. *n* = 2 mice per group. Fifteen images per mouse were acquired and quantified. Data are expressed as mean ± SEM, ***p < .001, ****p < .0001. One‐way analysis of variance test followed by Tukey's multiple comparisons test.

Marked increases in excitatory synapses and glutamate *N*‐methyl‐D‐aspartate receptors (NMDARs) cause dysregulation of glutamate homeostasis and are a major factor contributing to CIPN.^30^, ^31^ We previously discovered that astrocyte‐secreted Sparcl1 controls proper excitatory synapse formation that recruits NMDARs to the synapse and is required for synaptic plasticity.^20^ Moreover, more recently, we demonstrated that Sparcl1 is increased in neuropathic pain and can drive pathological pain through spinal cord astrocyte and NMDARs signaling.^22^ Because we have shown that activation of S1PR1 on astrocytes by S1P increased expression of Sparcl1 and astrocyte synapse interactions,^32^ we next analyzed the effect of Paclitaxel on Sparcl1 expression and determined the effect of S1PR1 antagonism. There was an approximately twofold increase in Sparcl1 intensity in the paclitaxel‐treated mice and FTY720 co‐administration profoundly reduced Sparcl1 expression (Figure 2A,B). This is consistent with the reversal of mechanical‐ and cold‐allodynia by this FTY720 regimen (Figure 1A,B). Moreover, confocal microscopy revealed that Sparcl1 is expressed by the majority of GFAP‐positive astrocytes and that FTY720 administration significantly reduced the expression of Sparcl1 in reactive astrocytes (Figure 2C).

**FIGURE 3:**
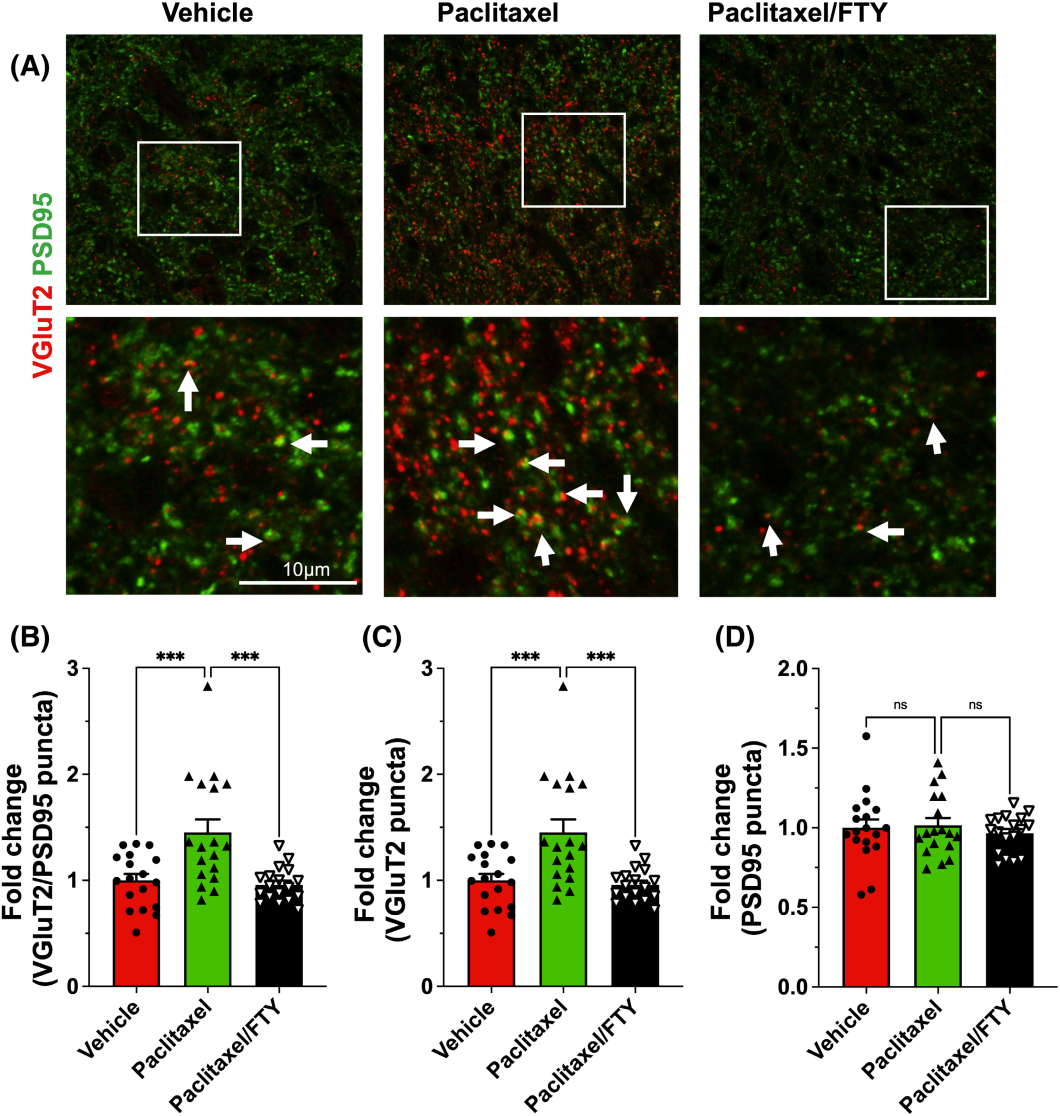
FTY720 suppresses paclitaxel‐induced excitatory synapses in naïve mice. Mice were treated with vehicle, paclitaxel without or with FTY720, as described in Figure 1. (A) Representative confocal images of the lamina I‐II area immunostained for VGluT2 (red) and PSD95 (green). Boxed area is zoomed in and shown in the lower panel. Excitatory synapses (arrow) are visualized as colocalized puncta (yellow) between VGluT2 and PSD95 puncta. Quantification of excitatory synapses (B), presynaptic terminal VGluT2 (C), and postsynaptic site PSD95 (D) as fold change compared to vehicle group. *n* = 3 mice per group. Eighteen images per group were acquired and quantified. Data are expressed as means ± SEM, ***p < .001. One‐way analysis of variance test followed by Tukey's multiple comparisons test.

As we have shown that Sparcl1 regulates VGluT2+ excitatory synapse formation and function^20^ and that VGluT2 but not VGluT1 is required for the proper synaptic transmission of the pain pathway and the development of neuropathic pain,^33^, ^34^ we analyzed the VGluT2 containing excitatory synapses in the lamina I‐II, the primary site of projection for C‐fiber and A δ‐fiber neurons in the DHSC of paclitaxel‐treated mice (Figure 3A). Interestingly, paclitaxel‐treated mice had a nearly 70% increase in excitatory synapses and a nearly 50% increase in presynaptic terminal VGluT2 at the peak pain period and FTY720 administration completely blocked this increase (Figure 3A–C). No changes were observed in PSD‐95, a postsynaptic scaffolding protein in excitatory neurons (Figure 3D). Taken together, these data suggest that targeting the S1P/S1PR1 axis suppresses Sparcl1 expression and excitatory synapses in CIPN.

### 3.2 FTY720 enhances paclitaxel efficacy to suppress TNBC progression and metastasis

**FIGURE 4:**
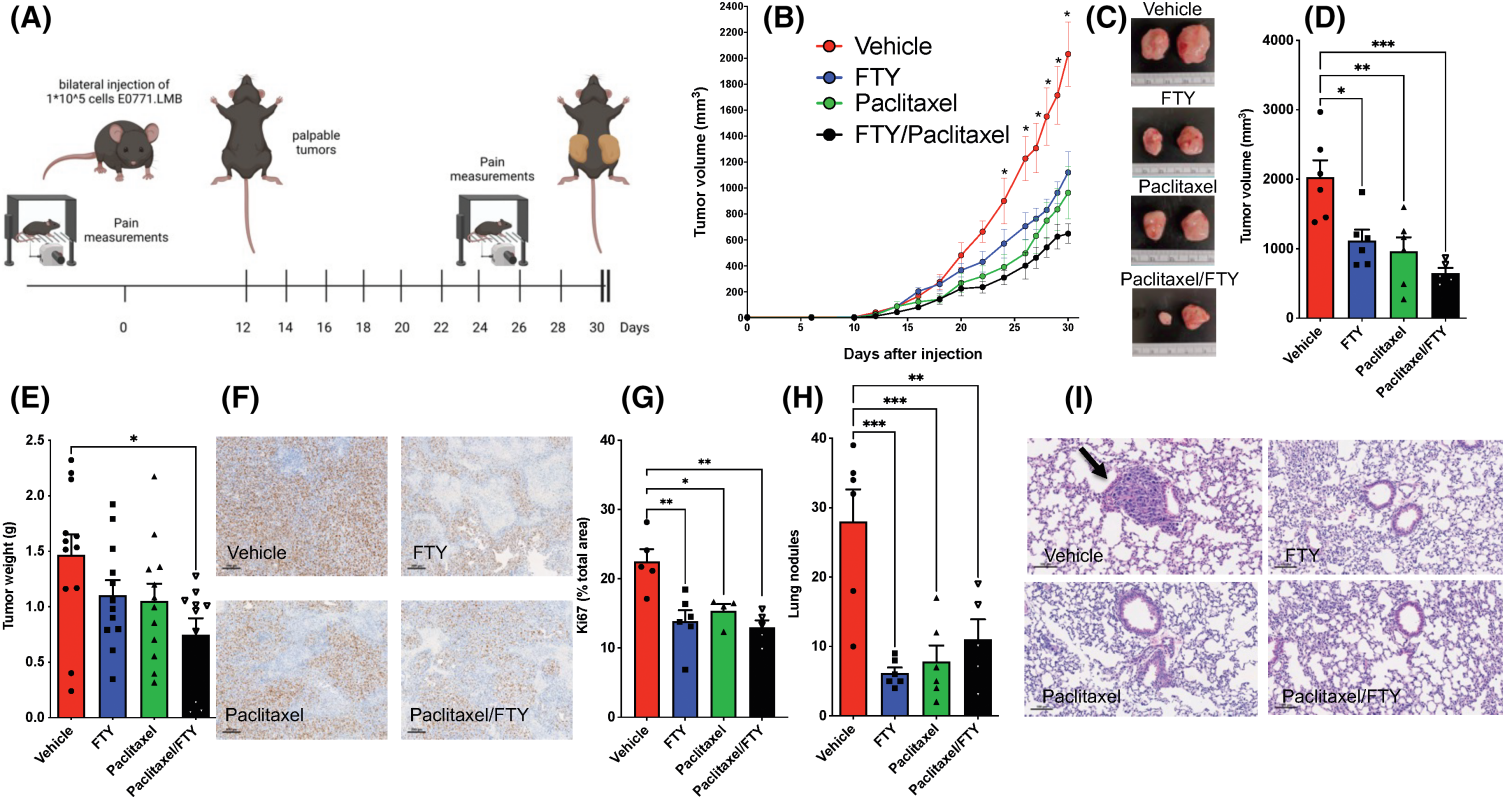
FTY720 enhances paclitaxel efficacy to reduce TNBC progression and metastasis. E0771.LMB breast cancer cells were implanted in the mammary fat pads of female mice and 12 days later, mice were treated with vehicles or FTY720 by gavage (0.3 mg/kg), paclitaxel (i.p. 10 mg/kg), or both every other day. *n* = 6, 6, 6, 5 mice. (A) Protocol for mice treatment and experiments. (B) Total tumor volumes were measured on the indicated days. *p ≤ .05 compared to drug‐treated groups (C–I) Mice were sacrificed on day 30. (C) Representative images of tumors. (D) Total tumor volumes. (E) Single tumor weights. (F and G) Tumor sections were stained with Ki‐67 antibody and proliferation determined by quantification of Ki67‐positive cells per high magnification field. *n* = 4–6 mice. Data are mean ± SEM. Scale bar: 100 μm. (H and I) Lung sections were stained with H&E. Black arrow indicates tumor nodule and lung metastasis determined by quantitation of visible metastatic nodules (H). Data are the means ± SEM. *p ≤ .05, **p ≤ .01, ***p ≤ .001. One‐way analysis of variance test followed by Tukey's multiple comparison test.

Similar to a previous study,^6^ we also found that paclitaxel‐induced neuropathic pain can be prevented by FTY720 administration (Figure 1A,B). However, these and most of the other previous studies on the prevention of CIPN by FTY720 have been carried out in naïve mice,^7^, ^8^, ^9^, ^10^, ^35^, ^36^, ^37^, ^38^ rather than in relevant cancer models. To overcome this deficiency, and to determine whether the presence of the tumor itself has an impact on pain, we examined the effects of FTY720 on TNBC tumor suppression and CIPN driven by paclitaxel in immunocompetent mice bearing E0771.LMB murine breast tumors (Figure 4A), a murine TNBC model that more accurately mimics human breast cancer.^39^ As expected, E0771.LMB cells produced large primary tumors in the mammary fat pad (Figure 4B,C). When tumors reached palpable size, mice were treated with paclitaxel (2 mg/kg), a clinically relevant dose of FTY720 (0.3 mg/kg), or both (Figure 4A). Paclitaxel or FTY720 alone suppressed tumor progression determined by decreases in primary tumor volumes and tumor weights. Co‐administration of both was slightly more effective than paclitaxel alone (Figure 4B–E). Consistent with the effects on tumor size, there was a significant decrease in tumor cell proliferation as determined by Ki‐67 staining after treatment with the drugs (Figure 4F,G). In agreement with previous studies,^15^, ^39^ E0771.LMB tumor cells spontaneously metastasized to the lungs. Lung metastatic spread was also greatly reduced by either FTY720 or paclitaxel or both as shown by macroscopic counts or histological analysis of metastatic nodules (Figure 4H,I).

### 3.3 FTY720 prevents CIPN in tumor‐bearing mice

**FIGURE 5:**
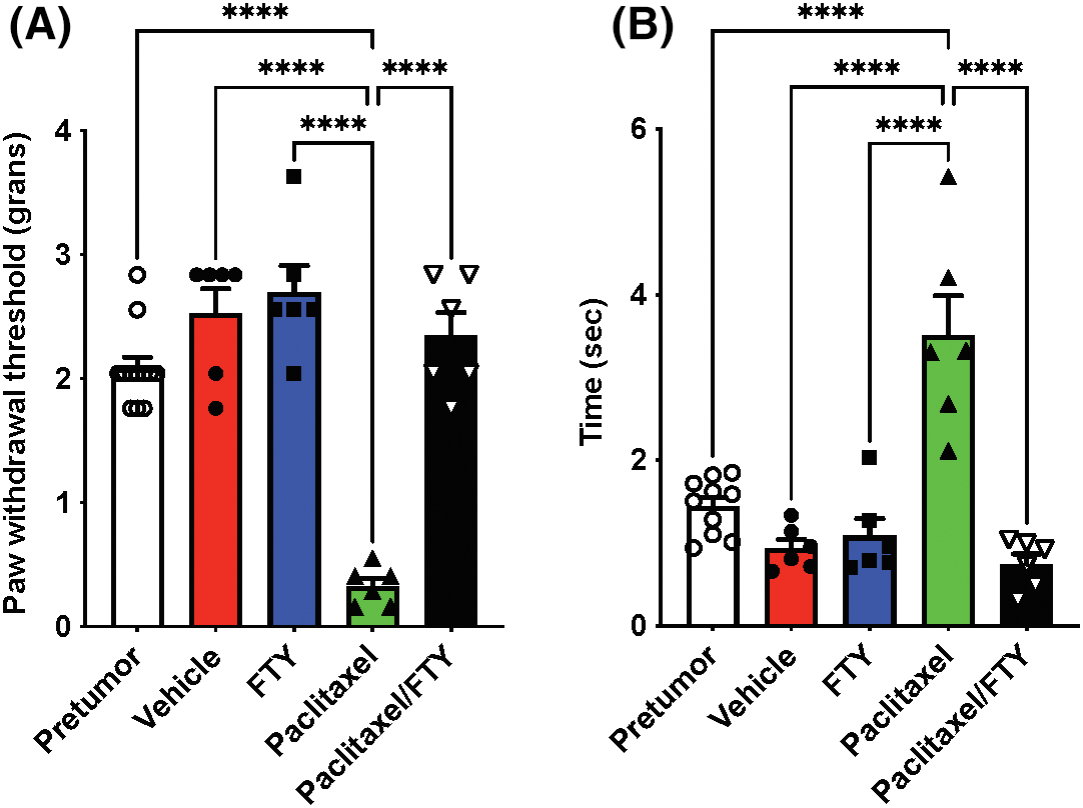
FTY720 reverses paclitaxel‐induced CIPN. E0771.LMB breast cancer cells were implanted in the mammary fat pads of female mice and 12 days later, mice were treated with vehicle or FTY720 by gavage (0.3 mg/kg), paclitaxel (i.p. 10 mg/kg), or both as described in Figure 4A. (A) Mechanical allodynia and (B) cold allodynia were determined on day 0 prior to tumor cell implantation or on day 25. *n* = 6 mice per group. For comparison, mechanical and cold allodynia are shown before tumor implantation (pre‐tumor). *n* = 10, 12 mice per group. Data are the means ± SEM. ****p ≤ .0001. One‐way analysis of variance test followed by Tukey's multiple comparison test.

Pain sensitivity was measured prior to E0771.LMB cell implantation and during the course of the experiment. Surprisingly however, the presence of tumor did not significantly increase neuropathic pain (compare baseline before tumor implantation to vehicle control with tumor in Figure 5A,B). Consistent with previous reports in naïve mice^6^, ^40^, ^41^ (and Figure 1), Paclitaxel increased mechanical‐allodynia as measured by reduced paw withdrawal threshold and cold‐allodynia in the acetone test in breast tumor‐bearing mice (Figure 5A,B). Importantly, FTY720 treatment not only had anti‐cancer effects (Figure 4), it also prevented CIPN induced by paclitaxel in these TNBC‐bearing mice (Figure 5A,B).

### 3.4 S1PR1 antagonism attenuates paclitaxel‐induced Sparcl1 expression and excitatory synapses in tumor‐bearing mice

**FIGURE 6:**
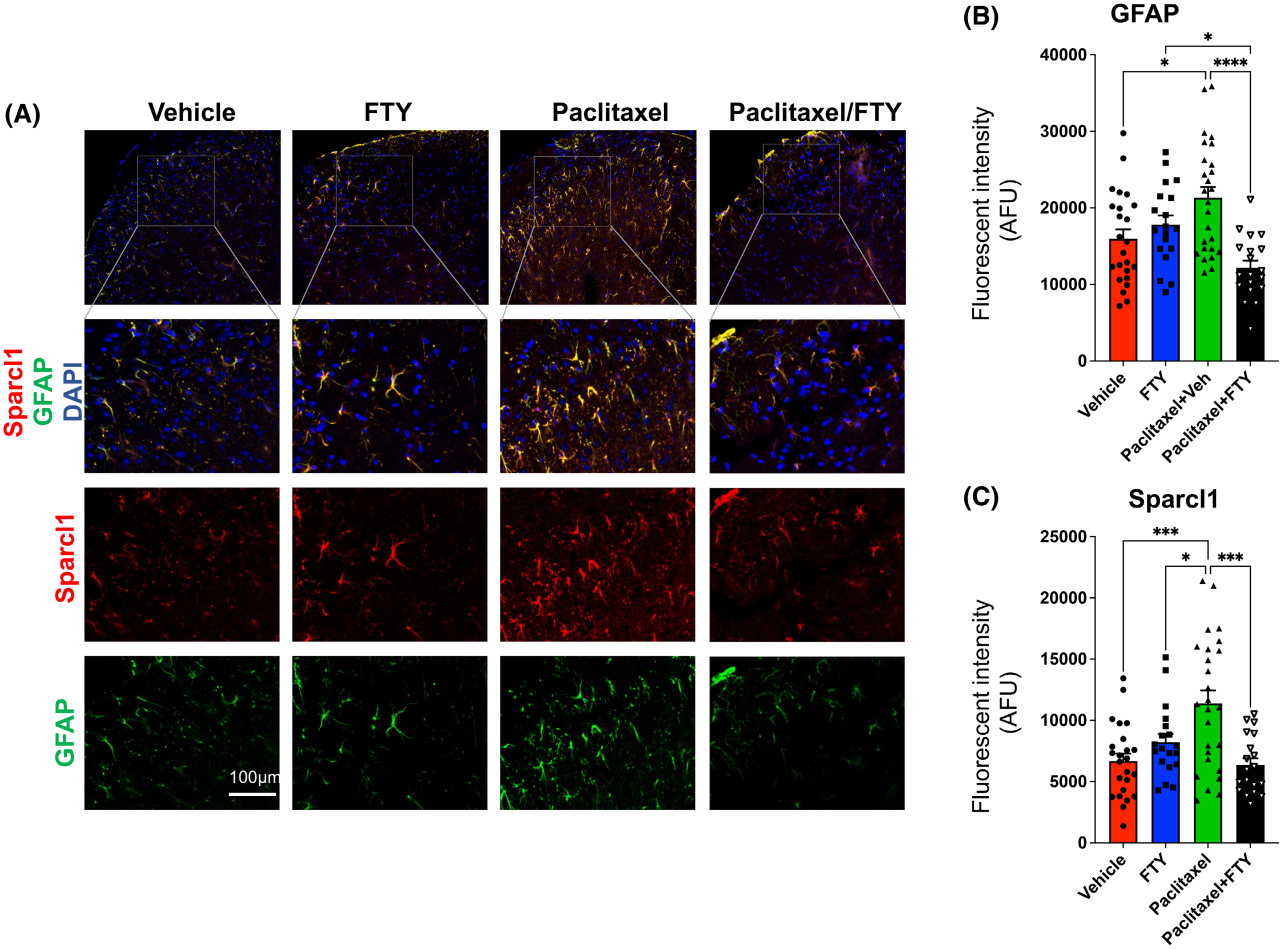
FTY720 suppresses paclitaxel‐induced astrocyte activation and Sparcl1 expression. E0771.LMB breast cancer cells were implanted in the mammary fat pads of female mice and 12 days later, mice were treated with vehicle, FTY720 (gavage 0.3 mg/kg), paclitaxel (i.p. 10 mg/kg), or both as described in Figure 4A. *n* = 4 mice per group. Lumbar DHSC was harvested from these mice on day 30. (A) Representative confocal images of the DHSC area immunostained for GFAP (green) and Sparcl1 (red). Quantification of GFAP (B) and Sparcl1 (C) fluorescence intensity plotted as integrated density per field. *n* = 4 mice per group. Nineteen to twenty‐six images per group were acquired and quantified. Data are expressed as means ± SEM, *p < .05, ***p < .001, ****p < .0001. One‐way analysis of variance test followed by Tukey's multiple comparisons test.

**FIGURE 7:**
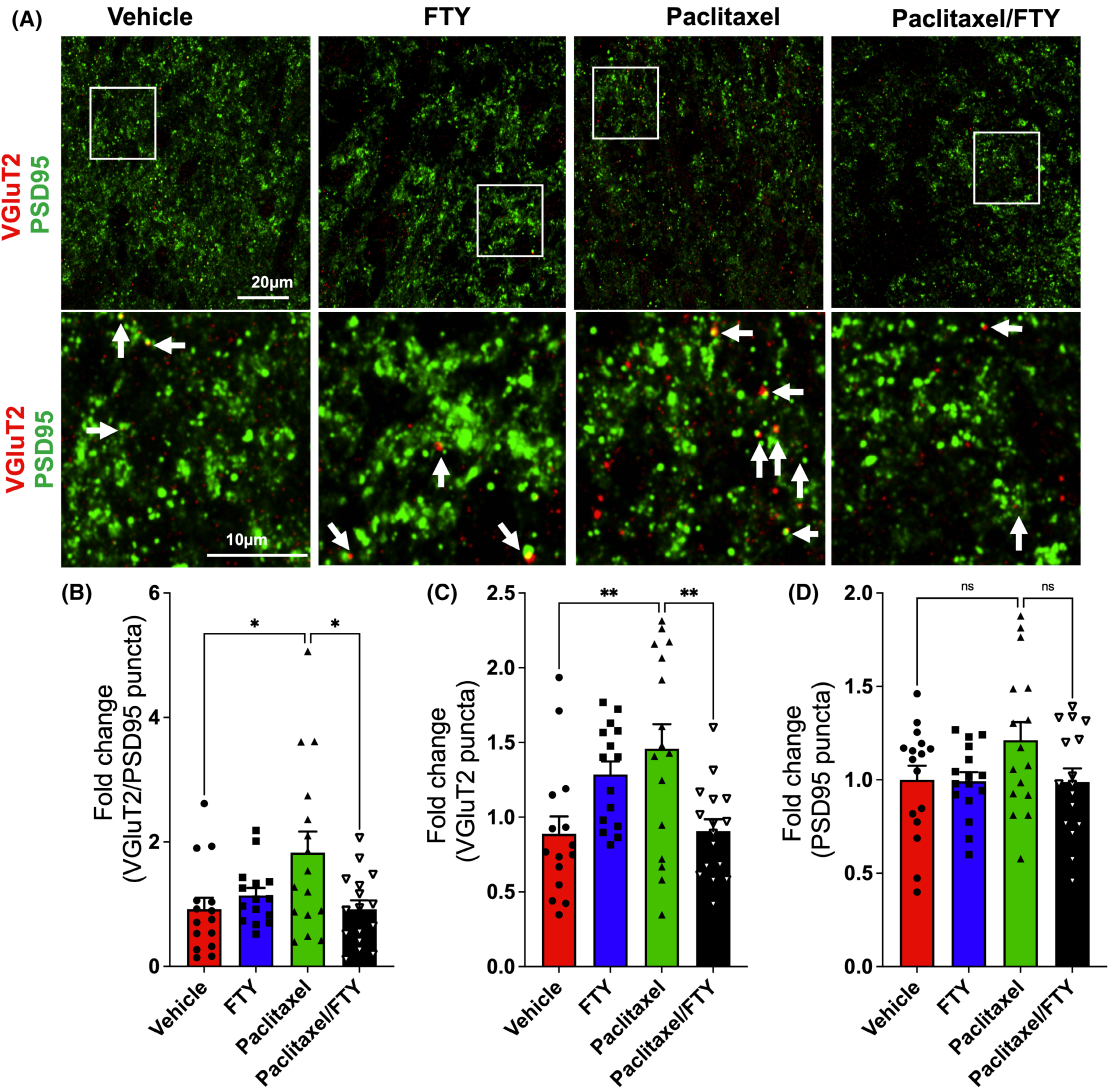
FTY720 suppresses paclitaxel‐induced excitatory synapses. E0771.LMB breast cancer cells were implanted in the mammary fat pads of female mice and 12 days later, mice were treated with vehicle, FTY720 (gavage 0.3 mg/kg), paclitaxel (IP 10 mg/kg), or both (*n* = 4) as described in Figure 4A. Lumbar DHSC was harvested from these mice on day 30. (A) Representative confocal images of the lamina I‐II areas immunostained for VGluT2 (red) and PSD95 (green). Boxed area is zoomed in and shown in the lower panel. Excitatory synapses (arrow) are visualized as colocalized puncta (yellow) between VGluT2 and PSD95 puncta. Quantification of excitatory synapses (B), presynaptic terminal VGluT2 (C), and postsynaptic site PSD95 (D) as fold change compared to vehicle group. *n* = 4 mice per group. Sixteen images per group were acquired and quantified. Data are expressed as means ± SEM, *p < .05, **p < .01. One‐way analysis of variance test followed by Tukey's multiple comparisons test.

Recent evidence suggests that cancer microenvironment and pain‐associated nerve signaling pathways are interdependent in such that enhanced pain stimuli may lead to poor cancer pathology.^42^ Although we did not find a significant effect of tumor itself on mechanical and cold allodynia in our breast cancer‐bearing CIPN mouse model (Figure 5A,B), it was of interest to examine how the astrogliosis and neuronal synapses will be impacted. Interestingly, in line with previous reports showing that after CIPN, astrogliosis appears to be more noticeable and persistent than microgliosis in the DHSC,^10^, ^28^, ^43^, ^44^ paclitaxel‐induced CIPN was associated with increased expression of GFAP in TNBC bearing mice and that FTY720 co‐application completely blocked the upregulation of GFAP expression (Figure 6A,B). Consistent with our findings in naive mice (Figure 2), paclitaxel treatment significantly increased expression of Sparcl1 in the DHSC of the lumbar region L4‐L6 and FTY720 co‐treatment completely prevented this upregulation in these TNBC‐bearing mice (Figure 6A,C). Similarly, we analyzed the VGluT2 containing excitatory synapses in the lamina I‐II, the primary site of projection for C‐fiber and Aδ‐fiber neurons in the DHSC of paclitaxel‐treated tumor‐bearing mice (Figure 7A,B). Paclitaxel‐treated mice showed about 80% increase in VGluT2/PSD95 colocalized synapses and FTY720 co‐treatment completely blocked this increase (Figure 7B). This is consistent with the reversal of mechanical and cold allodynia by this FTY720 regimen (Figure 5A,B). In addition, we found that while presynaptic VGluT2 puncta significantly increased (Figure 7C), there were no changes in postsynaptic PSD95 puncta numbers (Figure 7D), suggesting paclitaxel primarily affected presynaptic terminals without changing postsynaptic sites in breast cancer‐bearing mice. We also noticed that the baseline expression of Sparcl1 and particularly GFAP was much higher in TNBC‐bearing mice compared to naïve mice (compare vehicle groups between Figure 6 with Figures 1 and 2), which could be due to the presence of tumors.

## 4 DISCUSSION

Here we substantiate our previous finding that the S1P‐S1PR1 axis is a critical determinant in the development and maintenance of paclitaxel‐induced neuropathic pain.^6^ Consistent with the notion that spinal astrocytes contribute to the pathogenesis of paclitaxel‐induced painful neuropathy,^28^, ^29^ we showed that paclitaxel‐induced mechanical allodynia in naive and breast cancer‐bearing mice significantly increased levels of astrocyte‐secreted Sparcl1. In line with our previous observation that binding of S1P to astrocytic S1PR1 induced expression of Sparcl1,^32^ we found that targeting the S1P/S1PR1 axis with the FDA approved drug, FTY720/Fingolimod suppressed paclitaxel‐induced neuropathic pain and Sparcl1 expression. Our data support previous studies suggesting that Sparcl1 is important for the maintenance of neuropathic pain.^22^ It was found that deletion of Sparcl1 in mice suppressed nerve injury‐induced mechanical allodynia in the maintenance phase, but not the induction phase of neuropathic pain and its re‐expression in astrocytes caused the pain to return.^22^ Moreover, intrathecal injection of Sparcl1 induced persistent mechanical allodynia.^22^ Similar to our results, substantial increases of Sparcl1 have been observed in human CSF after painful neurosurgical procedures and in mouse CSF after nerve injury^22^ and after plantar incision in rats.^23^

We have previously shown that Sparcl1 facilitates the formation of Vesicular Glutamate Transporter 2‐positive (VGlut2‐positive) synapses by bridging neurexin‐1α (Nrx1α), a presynaptic component, and neuroligin‐1B (NL1B), a postsynaptic component, and recruits GluN2B‐containing NMDAR subunit to the thalamocortical synapses in the developing visual cortex to increase neuronal circuit plasticity.^20^ Indeed, Sparcl1‐induced mechanical pain was completely blocked by a GluN2B antagonist supporting the idea that Sparcl1 induces central sensitization and mechanical pain via regulation of GluN2B containing NMDARs in spinal cord dorsal horn neurons.^22^ Our results suggest that S1P/S1PR1 signaling enhances Sparcl1 expression that in turn plays critical roles in increasing VGluT2^+^ excitatory synapses in CIPN. Others showed that Sparcl1 promoted postoperative pain hypersensitivity by enhancing the neurexin1ß and neuroligin1‐mediated synaptic targeting of GluA1 subunits of AMPAR in spinal dorsal horns.^23^ Nevertheless, no changes were observed in AMPA‐induced currents in spinal dorsal horns neurons of Sparcl1 KO mice.^22^ Taken together, it seems that Sparcl‐1‐induced synaptic plasticity is important not only during development but also in the induction of neuropathic pain.

We also previously reported that activation of S1P/S1PR1 also induced expression of astrocyte‐secreted synaptogenic factor Thrombospondin‐4 (TSP‐4), a member of the thrombospondin family of extracellular matrix proteins.^32^ In addition to Sparcl1, emerging evidence suggests that TSP‐4 also contributes to spinal sensitization and neuropathic pain states.^45^, ^46^ TSP‐4 is also mainly expressed in astrocytes and studies in animal models have demonstrated that increased expression of TSP‐4 correlates with the development of neuropathic pain.^45^ Moreover, intrathecal injection of TSP‐4 increased miniature excitatory postsynaptic currents and caused neuropathic pain.^44^ It is thus tempting to speculate that targeting the S1P/S1PR1 axis with FTY720 might also reduce TSP‐4 levels in CIPN.

The symptoms of paclitaxel‐induced CIPN, loss of sensation, and tingling sensation, usually appear days after the treatment and may persist for weeks, months, or even years after drug termination.^47^ CIPN reduces the quality of life of cancer survivors and can interfere with effective anticancer treatment.^47^, ^48^ Dose reduction or treatment termination is often the only recourse for CIPN and there are no preventive or curative interventions.^47^, ^48^, ^49^ The current symptomatic therapies, antidepressants, and morphine, have not shown consistent effectiveness in most patients and also have serious side effects and potential for drug abuse.^49^ Therefore, new therapies that can prevent, alleviate, and/or reverse the development of CIPN are needed. In this regard, we previously found that several functional S1PR1 antagonists including ponesimod and siponimod now in clinical use for other indications,^50^ and KRP‐203, as well as competitive S1PR1 antagonists had similar efficacy and potency as FTY720 in mouse models of traumatic nerve injury‐induced neuropathic pain.^8^ Because these S1PR1 modulators have limited S1PR3 activity, these results challenged the involvement of S1PR3 in the beneficial effects of FTY720.^51^ Altogether, these findings support the development of S1PR1 antagonists, rather than agonists, as a useful category of non‐narcotics to relieve CIPN.^10^

Our study has some limitations. The quantification of astrocyte activation, Sparcl1 expression, and excitatory synapse formation were based on confocal fluorescence microscopy of brain slices. Although this approach has been extensively used,^20^, ^52^, ^53^, ^54^ it may be error‐prone and subjective. Another potential drawback is that although we have shown mechanistically that paclitaxel‐induced activation of astrocytes contributes to neuropathic pain by secretion of Sparcl1, we did not establish the critical role of Sparcl1 by reducing its levels with a neutralizing antibody. Even though it was suggested that neutralizing secreted Sparcl1 with antibodies may be beneficial for reducing acute and chronic pain,^22^ this is not a good strategy for treating CIPN in TNBC patients as data from the TCGA PanCancer Atlas indicate that high Sparcl1 mRNA expression correlates with better survival (https://www.proteinatlas.org/ENSG00000152583‐Sparcl1/pathology/breast+cancer). Moreover, further studies are needed to examine the translational potential of our findings, by assessing levels of Sparcl1 in CSF from TNBC patients treated with paclitaxel.

Consistent with our findings, oral administration of clinically relevant doses of FTY720 has been shown to reduce tumor growth progression and metastases in several TNBC mouse models^13^, ^24^, ^55^ and inhibits expansion of breast cancer stem cells.^56^ Importantly, FTY720 treatment also enhanced the efficacy of numerous chemotherapeutic agents, such as paclitaxel (Figure 4), doxorubicin,^57^ and trastuzumab.^25^ Several mechanisms have been suggested to explain the anticancer activities of FTY720. The active form FTY720‐P, a functional antagonist of S1PR1, can restrain tumor growth via various S1PR1‐dependent mechanisms: (1) suppressing proapoptotic Bim and enhancing pro‐survival Mcl‐1 proteins^58^; (2) disrupting the S1P/SphK1/S1PR1 positive feedback loop for sustained NF‐kB and STAT3 activation within the breast tumor microenvironment,^13^ crucial for TNBC progression; (3) Nuclear FTY720‐P also acts as a potent inhibitor of class I HDACs, and sensitizes breast cancer cells to tamoxifen therapy.^24^ In addition, the pro‐drug FTY720 (the unphosphorylated form) is a strong activator of protein phosphatase 2A (PP2A), a heterotrimeric serine/threonine phosphatase that counteracts kinase‐driven survival signaling pathways, including MEK and AKT.^57^ Notably, diminished PP2A activity is a common occurrence in breast cancer and may predict sensitivity to FTY720.^57^ The unphosphorylated FTY720 also impedes and promotes the proteasomal degradation of SphK1, which is elevated in breast cancer, correlating with unfavorable prognosis and drug resistance.^59^

Our work suggests that targeting the S1P/S1PR1 axis with FTY720 is a multipronged approach to bolster the anti‐cancer effects of paclitaxel in TNBC while concurrently mitigating CIPN. FTY720 presents numerous advantages. It is an orally bioavailable medication that has already garnered approval for human use. With favorable pharmacokinetics and a lengthy half‐life, it ensures sustained therapeutic effects. Moreover, FTY720 exhibits low toxicity and has the capability to accumulate in the CNS. There is no indication of tolerance or abuse associated with its usage. Notably, it also demonstrates the supplementary benefit of impeding tumor growth and metastasis in preclinical investigations, particularly in TNBC. Furthermore, the existence of next‐generation S1PR1‐targeted agents like Ozanimod (Zeposia, Celgene) and Siponimod (Mayzent), FDA‐approved drugs for the treatment of multiple sclerosis,^50^ suggests promising avenues for further advancements in this field. Hence, our results establish a scientific rationale to develop S1PR1‐targeted therapeutic agents for the treatment of TNBC and CIPN that may improve patient survival outcomes.

## AUTHOR CONTRIBUTIONS

S.K.S. and S.S. designed the study and wrote the manuscript. S.K.S, C.W., C.D.G., R.D.R.B., and C.T. performed experiments. S.S. and D.S. provided support for the study. S.S and D.S. provided guidance on the experimental design. All authors reviewed and approved the final manuscript.

## FUNDING INFORMATION

The funding source did not have any role in study design, data collection, data analyses, interpretation, or writing of report.

## DISCLOSURES

All the authors declare no potential conflicts of interest.

## Data Availability

All data needed to evaluate the conclusions in the paper are presented in the paper. Additional data are available upon request from Dr. Sandeep Singh.
